# Botulinum Toxin A in Poststroke Oromandibular Dystonia: Case Reports

**DOI:** 10.1155/crnm/6373183

**Published:** 2026-02-09

**Authors:** Elena Brevi, Davide Villa, Davide Dalla Costa, Luciana Sciumé, Andrea Cattelan, Dante Facchetti, Giovanna Beretta, Maria Sessa

**Affiliations:** ^1^ School of Specialization in Physical Therapy and Rehabilitative Medicine, The University of Milan, Milan, Italy, unimi.it; ^2^ Department of Neurology and Stroke Unit, ASST Grande Ospedale Metropolitano Niguarda, Milan, Italy, ospedaleniguarda.it; ^3^ Department of Rehabilitation Medicine and Neurorehabilitation Unit, ASST Grande Ospedale Metropolitano Niguarda, Milan, Italy, ospedaleniguarda.it

## Abstract

**Background:**

Poststroke oromandibular dystonia (OMD) is a rare and challenging to treat form of dystonia. Botulinum toxin A (BoNT‐A) is a standard treatment in other etiologies of OMD, but literature concerning a long‐term potential beneficial role in posthemorrhagic stroke OMD is scarce.

**Cases:**

We present a case series of three patients with acute onset of multiple cranial nerve impairment and contralateral hemiparesis consequent to pontine hemorrhage. After a few months, all patients developed jaw‐closing OMD (JC‐OMD), conditioning a reduced mouth opening. Ultrasound‐guided BoNT‐A administration was started, and after 4 weeks, every patient showed clinical improvement. The results were maintained up to 10 years of follow‐up, and no adverse event was reported.

**Conclusions:**

Poststroke OMD is an underestimated condition. Our cases provided data of safety and efficacy of BoNT‐A treatment up to 10 years of follow‐up. Further studies are warranted to establish etiopathological mechanisms and standardized treatments for poststroke JC‐OMD.

## 1. Introduction

Oromandibular dystonia (OMD) is a chronic disorder characterized by sustained muscle contraction involving mouth, jaw, and tongue [1]. The surface and needle electromyographic hallmarks typically documented in dystonia include (i) abnormally prolonged motor unit discharges, (ii) cocontraction of agonist and antagonist muscle groups, and (iii) involuntary recruitment of anatomically adjacent muscles [2]. This electrophysiological phenotype reflects a sustained or intermittently increased central motor drive to α‐motor neurons, thereby differentiating spastic dystonia from spasticity, in which hyperexcitability is primarily phasic and dependent on stretch‐reflex mechanisms [3].

Based on muscular involvement and dystonic pattern, OMD is classified into six different subtypes: jaw closing, jaw opening, jaw deviation, jaw protrusion, lingual, and lip [4]. Jaw‐closing OMD (JC‐OMD) is characterized by restricted mouth opening due to tetanic contraction of masseter, temporalis, and medial pterygoid muscles, leading to masticatory disturbances, dysphagia, dysarthria, upper airway obstructions, and temporomandibular joint dislocations [1]. The majority of these symptoms negatively impact daily activities.

Based on etiology, OMD is classified as idiopathic or acquired [1] and within the acquired forms, stroke is considered a rare cause of OMD [5]. Little data exist in literature regarding therapeutic strategies for this particular form, including botulinum toxin A (BoNT‐A), which is already accepted as a standard treatment in other etiologies of OMD [6, 7]. We present a case series of three patients who developed JC‐OMD after pontine hemorrhagic stroke and showed clinical improvement with BoNT‐A chemodenervation. The therapeutic results were sustained at the 10‐year follow‐up in two of the three patients, whereas for the third patient only a 3‐month observation period was available.

## 2. Case Series

Three patients with acute onset of multiple cranial nerve impairment and contralateral hemiparesis (Table 1) were referred for specialist follow‐up. Magnetic resonance imaging (MRI) showed pontine hematoma secondary to arteriovenous malformation in Patient 1 and cavernomatosis in Patients 2 and 3 (Figure 1). Neurosurgical hematoma evacuation was performed, with partial recovery following treatment. Due to clinical stability, the three patients were transferred to the rehabilitation unit. Post 1 month and 3 months, one patient and two patients, respectively, developed JC‐OMD, experiencing the inability to open their mouths over 15 mm in Patient 1, 17 mm in Patient 2, and a complete inability in Patient 3. These parameters were obtained by measuring maximum interincisal opening (MIO) [8].

**Table 1:** Clinical summary of the case reports: age, sex, comorbidity, pontine hemorrhage etiology, clinical features, and MIO before and after treatment with BoNT‐A and its posology.

	**Patient 1**	**Patient 2**	**Patient 3**

Age	52	34	56
Sex	Male	Female	Female
Comorbidity	Hypertension	Congenital factor VII deficiency	Hypertension
Pontine hemorrhage etiology	AVM rupture	Cavernoma rupture	Cavernoma rupture
Clinical features	Right V, VI, VII, and VIII cranial nerves deficiency and contralateral hemiparesis	Right V and VII cranial nerves deficiency and contralateral hemiparesis	Left VI and VII cranial nerves deficiency and contralateral hemiplegia
Onset of OMD after pontine hemorrhage (days)	92	34	30
Muscle involvement in OMD	Right masseter and temporalis muscles	Right masseter and temporalis muscles	Left masseter and temporalis muscles
BoNT‐A injected in temporalis muscle (U)	20	25	40
BoNT‐A injected in masseter muscle (U)	20	25	60
MIO before treatment (mm)	15	17	0
MIO at 1 month of follow‐up (mm)	20	48	10
MIO at 3 months of follow‐up (mm)	20	48	10
MIO at 1 year of follow‐up (mm)	20	48	/
MIO at 5 years of follow‐up (mm)	25	48	/
MIO 10 years of follow‐up (mm)	35	48	/

*Note:* AVM, arteriovenous malformation; OMD, oromandibular dystonia; BoNT‐A, botulinum toxin A; U, units; mm, millimeters.

Abbreviation: MIO, maximum interincisal opening.

Figure 1Magnetic Resonance Imaging (MRI) showing a voluminous pontine cavernoma with signs of recent bleeding and surrounding edema in Patient 3, as seen on sagittal T2‐weighted and fluid‐attenuated inversion recovery (FLAIR) images (a) and axial susceptibility‐weighted imaging (SWI) (b).

**Figure:**
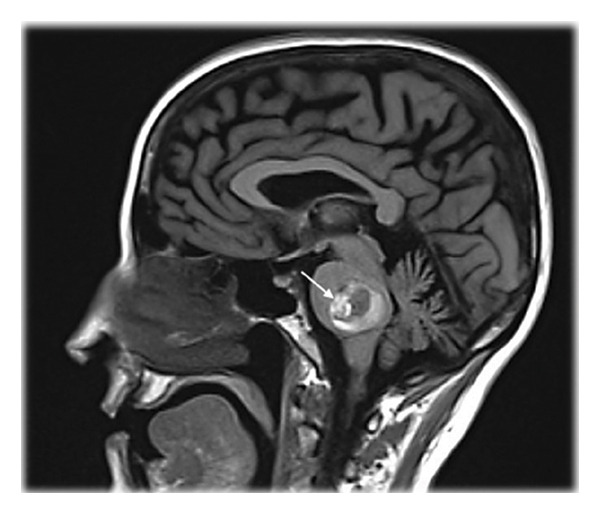
(a)

**Figure:**
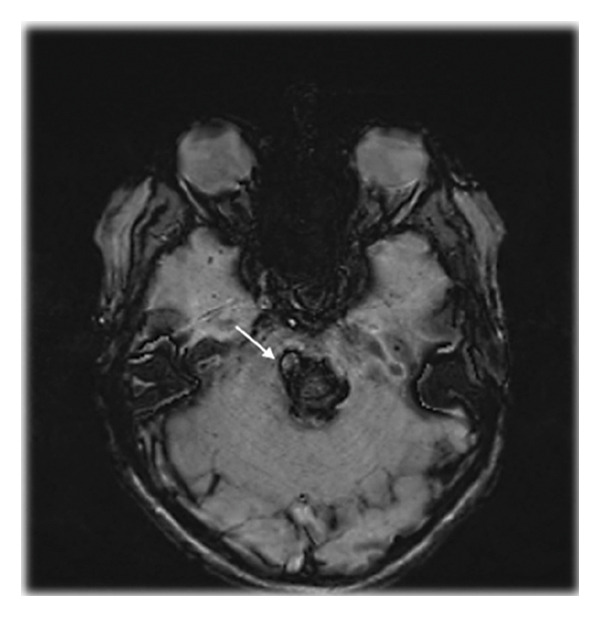
(b)

Concentric needle electromyography (EMG) documented persistent firing of motor unit action potentials (MUAPs) at rest and when patients were asked to open the mouth, in jaw‐closing muscles (masseter, temporalis, and medial pterygoid) and in jaw‐opening muscles (geniohyoid, omohyoid, and the anterior belly of the digastric), which exhibited voluntary activity also at rest; right side in Patient 1 and 2, and left side in Patient 3.

This pattern of cocontraction of agonist and antagonist muscles and also the prolonged motor unit firing usually occurs in dystonia as described by Rothwell [9].

Ultrasound‐guided BoNT‐A injections were performed with onabotulinumtoxinA (Botox) at a concentration of 100 units (U) diluted in 1 mL of saline. A 32‐gauge needle was used to minimize tissue trauma.

Injections were administered with a dose of 20 U + 20 U in Patient 1, 25 U + 25 U in Patient 2, and 60 U + 40 U in Patient 3 in two sites in masseter and temporalis muscles involved. The first two patients enrolled in the study are undergoing regular follow‐up with botulinum toxin administration every 6 months at the same dosage. The third patient completed only a 3‐month follow‐up because he subsequently underwent an early neurosurgical nerve transposition procedure and, therefore, no longer required further treatment at our institution.

Afterwards, all patients underwent cycles of physiotherapy that included mouth‐opening exercises using a helical mouth opener for 10 min every 2 h throughout the 2‐month recovery period, demonstrating good compliance. The patient was instructed to place the narrower end of the helical device between the upper and lower incisors and then to rotate the device clockwise using the dedicated handle.

They were advised to continue its use after discharge.

After 1 month (Table 1), all three patients showed clinical improvement. The MIO obtained was 20 mm for Patient 1, 10 mm for Patient 3, and Patient 2 reached a complete oral opening (MIO 48 mm) [8]. Accordingly, EMG documented a general reduction of rest activity in muscles involved. The results of initial therapy were maintained during the 10‐year follow‐up for Patient 2 and three months for Patient 3. Patient 1 showed further improvement by achieving an oral opening of 35 mm during the 10‐year follow‐up (Table 1). No adverse effects were reported. Patient satisfaction was evaluated with a patient‐reported outcome measure (PROM) employing a five‐point Likert scale, which yielded the maximum score for Patients 1 and 2 and a score of 3 for Patient 3.

## 3. Discussion

The present case series documented therapeutic success of BoNT‐A chemodenervation associated with physiotherapy in three patients with JC‐OMD developed after a pontine hemorrhage. Our results are consistent with other studies in literature regarding OMD secondary to different etiologies [1]. However, few studies investigated the potential efficacy of BoNT in JC‐OMD secondary to hemorrhagic stroke [10], where this is rarely considered a possible cause of JC‐OMD. All three patients showed a significant improvement of clinical parameters, including the possibility to eat, speak, and perform oral hygiene, following a few weeks of BoNT injection. This result confirms the potential role of BoNT‐A use in treating in poststroke OMD [11–14].

The pathophysiological mechanisms underlying the development of OMD are generally unknown, with the possibility of different pathways being involved. In other cases of poststroke OMD, the primary site of the lesions was in the cerebellum. A circuit‐level explanation of this phenomenon could derive from the lesion of the cerebello–thalamo–cortical network that could lead to deficiency of the GABAergic inhibition on the primary motor cortex in dystonic patients [15, 16]. Contrary to this, in our cases, the primary site of the lesion was the pons. The bilateral synchronic movements of the masticatory muscles are coordinated by the hypothetical central pattern generator for jaw movements in the pontine reticular formation [13]. Notably, any damage involving the corticonuclear tract and the pontine motor nucleus of trigeminal nerve could potentially lead to an insufficient coordination of the antagonistic inputs, resulting in an insufficient inhibition of jaw closers on jaw opening and thus in spasmodic activity of masseter, temporalis, and medial pterygoid muscles. However, the reason for JC‐OMD not occurring in every pontine lesion is still unknown.

In conclusion, JC‐OMD secondary to stroke is an underestimated condition and symptomatic treatment with BoNT‐A chemodenervation associated with physiotherapy demonstrated clinical efficacy. In addition, our long‐term data provided safety and persistent efficacy for up to 10 years in two patients. Further studies are required to understand etiopathological mechanisms and establish protocols for treating poststroke JC‐OMD.

## Author Contributions

Elena Brevi: conception, organization, execution, writing of the first draft, and review and critique.

Davide Villa: conception, organization, execution, writing of the first draft, and review and critique.

Davide Dalla Costa: organization, execution, and review and critique.

Luciana Sciumé: organization and review and critique.

Andrea Cattelan: organization and review and critique.

Dante Facchetti: organization, execution, and review and critique.

Giovanna Beretta: organization and review and critique.

Maria Sessa: organization and review and critique.

## Funding

No specific funding was received for this work. Open access publishing was facilitated by Azienda Socio Sanitaria Territoriale Grande Ospedale Metropolitano Niguarda, as part of the Wiley–SBBL agreement.

## Disclosure

All authors have read the manuscript and agreed to conditions noted on the Authorship Agreement Form. They also confirm that the data have not been previously published and that the manuscript is not under simultaneous consideration by another journal. They take full responsibility for the data, the analyses and interpretation, and the conduct of the research. They have full access to all the data, and they have the right to publish them separate and apart from any sponsor.

## Ethics Statement

Approval for this study was granted by the ethical standards committee of ASST Grande Ospedale Metropolitano Niguarda.

Written informed patient consent was obtained before submitting this work.

We confirm that we have read the Journal’s position on issues involved in ethical publication and affirm that this work is consistent with those guidelines.

## Conflicts of Interest

The authors declare no conflicts of interest.
